# Sex-Related Differences of Cortical Thickness in Patients with Chronic Abdominal Pain

**DOI:** 10.1371/journal.pone.0073932

**Published:** 2013-09-05

**Authors:** Zhiguo Jiang, Ivo D. Dinov, Jennifer Labus, Yonggang Shi, Alen Zamanyan, Arpana Gupta, Cody Ashe-McNalley, Jui-Yang Hong, Kirsten Tillisch, Arthur W. Toga, Emeran A. Mayer

**Affiliations:** 1 Gail and Gerald Oppenheimer Family Center for Neurobiology of Stress and Pain and Interoception Network (PAIN) Repository, Department of Medicine, David Geffen School of Medicine, University of California Los Angeles, Los Angeles, California, United States of America; 2 Human Performance and Engineering Laboratory, Kessler Foundation Research Center, West Orange, New Jersey, United States of America; 3 Department of Biomedical Engineering, New Jersey Institute of Technology, Newark, New Jersey, United States of America; 4 Laboratory of NeuroImaging (LONI), University of California Los Angeles, Los Angeles, California, United States of America; 5 Ahmanson-Lovelace Brain Mapping Center, University of California Los Angeles, Los Angeles, California, United States of America; 6 University of Michigan, Ann Arbor, Michigan, United States of America; West China Hospital of Sichuan University, China

## Abstract

**Background & Aims:**

Regional reductions in gray matter (GM) have been reported in several chronic somatic and visceral pain conditions, including irritable bowel syndrome (IBS) and chronic pancreatitis. Reported GM reductions include insular and anterior cingulate cortices, even though subregions are generally not specified. The majority of published studies suffer from limited sample size, heterogeneity of populations, and lack of analyses for sex differences. We aimed to characterize regional changes in cortical thickness (CT) in a large number of well phenotyped IBS patients, taking into account the role of sex related differences.

**Methods:**

Cortical GM thickness was determined in 266 subjects (90 IBS [70 predominantly premenopausal female] and 176 healthy controls (HC) [155 predominantly premenopausal female]) using the Laboratory of Neuro Imaging (LONI) Pipeline. A combined region of interest (ROI) and whole brain approach was used to detect any sub-regional and vertex-level differences after removing effects of age and total GM volume. Correlation analyses were performed on behavioral data.

**Results:**

While IBS as a group did not show significant differences in CT compared to HCs, sex related differences were observed both within the IBS and the HC groups. When female IBS patients were compared to female HCs, whole brain analysis showed significant CT increase in somatosensory and primary motor cortex, as well as CT decrease in bilateral subgenual anterior cingulate cortex (sgACC). The ROI analysis showed significant regional CT decrease in bilateral subregions of insular cortex, while CT decrease in cingulate was limited to left sgACC, accounting for the effect of age and GM volume. Several measures of IBS symptom severity showed significant correlation with CT changes in female IBS patients.

**Conclusions:**

Significant, sex related differences in CT are present in both HCs and in IBS patients. The biphasic neuroplastic changes in female IBS patients are related to symptom severity.

## Introduction

Neuroplastic gray matter (GM) changes have been reported in a wide range of chronic pain conditions, including both visceral and somatic pain syndromes [1], [2]. Based on the similarities of brain regions affected, and the resolution of structural brain changes following successful therapy of peripheral causes of chronic somatic pain reported in a few studies [3], [4], it has been suggested that the observed changes are the consequence, and not the cause of the respective pain syndrome. However, generally agreed upon peripheral causes of pain have not been identified in the majority of persistent pain syndromes (e.g. irritable bowel syndrome [IBS], fibromyalgia [FM], painful bladder syndrome/interstitial cystitis [PBS/IC]), and the neurobiological mechanisms underlying the observed neuroplastic changes remain unknown. More than 30 publications on this topic have appeared in the literature to date, yet the small sample size, heterogeneous populations, lack of control for pain medications patients were on, lack of control for sex, and difference in analytic methods, have limited the ability to make definitive statements about the cause and relationship of such changes to the clinical presentation. The majority of studies have reported reduction of GM in poorly defined subregions of insular and anterior cingulate cortices, thalamus and prefrontal cortex. However, some studies have reported both increases and decreases in GM [3], [5], or increases only [6]. Some of the observed disease related differences were no longer seen when differences in anxiety and depression were taken into consideration [3].

Irritable bowel syndrome (IBS) is the most common visceral pain disorder, which is more common in women. Affected patients present with chronically recurrent abdominal pain and discomfort associated with altered bowel habits, and increased levels of anxiety [7], symptom-related anxiety [8], and catastrophizing [9]. IBS symptoms often overlap with symptoms of other persistent pain syndromes and with disorders of mood and affect [10], and such comorbidites are more common in female patients [11]. Women are also more likely to develop IBS-like symptoms following a gastroenteric infection [12]. Sex related differences in evoked and resting state brain activity has been reported in IBS patients, including differences in an emotional arousal circuit [13], [14], [15]. Based on this converging evidence about sex related differences in IBS pathophysiology, we hypothesized the presence of sex related differences in GM changes. Considerable sex differences in brain structure, function and biochemistry have been reported in healthy individuals, which may in part be due to organizational and influential effects of sex hormones [16]. Two studies with female IBS patients only, have reported GM differences compared to HC subjects. While one of these studies showed both disease and symptom related GM reductions (both in cortical thickness [CT] and volume) in prefrontal and insular cortices in 11 patients [5], the other showed widespread reductions in cortical and subcortical areas in a sample of 55 patients, with a significant number of these changes being related to symptoms of anxiety and depression [3]. The two studies also differed in brain regions that showed greater regional GM, with the former showing increased GM volume in the hypothalamus, and the other in pregenual cingulate, orbofrontal cortex and posterior insula/somatosensory cortex.

To assist in our large-scale analyses we employed the LONI pipeline, a graphical workflow environment which allows users to describe executable tools in a graphical user interface and create processing modules as nodes in a graph representing the complete computational protocol [17], [18]. We aggregated data across 14 structural neuroimaging studies at UCLA conducted between August, 2006 and April, 2011, and examined group differences in regional CT in a large sample of 266 subjects (90 IBS, 176 HCs). Specifically, we aimed to accurately estimate the localized increases and decreases of widespread CT changes across the entire cortex, as well as track morphometric changes in key subregions of interest, e.g., the insula and anterior cingulate cortex. We show sex related CT differences in both disease and HC groups, and demonstrate that the observed neuroplastic changes in somatosensory and sgACC in female patients are correlated with clinical measures of symptom severity, with only limited correlations with symptom duration. The Pipeline environment was chosen because it provides two important benefits – the complete pipeline protocols can be openly disseminated as XML workflows (which facilitates results reproduction), and this infrastructure enables integration of heterogeneous software tools and diverse data from multiple studies [19], [20]. We decided to use CT in favor of grey matter volume and grey matter density as the structural index of grey matter because CT provides unsurpassed spatial resolution (vertex level analysis) [21].

## Methods

### Data & Subjects

T1-weighted brain images were aggregated from 14 University of California Los Angeles (Office of the Human Research Protection Program) Institutional Review Board approved structural imaging studies conducted at the Oppenheimer Family Center for Neurobiology of Stress as of December 31, 2011. Data is available on our Pain/Loni website (http://pain.loni.ucla.edu/) as part of the Pain and Interoception Network (PAIN) repository. Total 328 (IBS = 107, HC = 221) subjects were included in the current analysis. The exclusion criteria removed any subject who 1) tested positive for drug abuse; 2) had other medical problems other than IBS; 3) had an ongoing psychiatric illness as determined by the Mini International Neuropsychiatric Interview (MINI) [22]; 4) was engaged in excessive physical exercises (more than 8 hours/week of continuous exercise such as marathon runners or triathlon athletes) or 5) were taking medications which may interfere with CNS such as full dose antidepressants (including SSRI, NSRIs), sedatives or anxiolytics, and opioids. However, as the majority of recruited IBS patients are taking low dose tricyclic antidepressants, it would have been impossible to perform the study, if we excluded such subjects. As in our previous studies, we have therefore allowed patients on tricyclic antidepressants (TCAs) into the current study provided they were taking low doses of the drug (10–50 mg), and had been on a stable dose for at least 3 months.

All subjects gave their written consent before scanning. Diagnosis of IBS was made by history and clinical examination using the ROME II [23] symptom criteria based on assessment by a gastroenterologist or nurse practitioner trained in the diagnosis of functional bowel disease. Subjects with all subtypes of bowel habit (constipation, diarrhea, mixed, and unspecified) were included in the analysis. Following a quality control, a total of 266 subjects were analyzed, after removing the subjects with failing brain surface extraction and reconstruction, or sub-optimal registration into the ICBM (International Consortium of Brain Mapping) atlas space [24]. An experienced neuroanatomist rated each brain surface regarding the quality of surface reconstruction and accuracy of vertex labeling on a scale of 0 to 1 (0 = completely unacceptable; 1 = perfectly reconstructed and labeled). A threshold of 0.7 was used as a criterion for a subject to be included in the final analysis. Refer to **Figure S1** for an example surface with rating score provided.

### Clinic Assessment

**Table 1** provides the demographics of the IBS patients. Clinical parameters for the 266 subjects are in **Table 2**. Bowel Symptom Questionnaire (BSQ; [25]) were used to determine the overall severity of gastrointestinal symptoms during the past week (on a scale of 0 to 20; BSQ Overall), severity of abdominal pain during the past week (on a scale of 0 to 20), usual symptom severity during the past week (1 = none; 2 = mild; 3 = moderate; 4 = severe; 5 = very severe) and onset age of the symptom. Coping Strategies Questionnaires (CSQ; [26]) was administered to measure the catastrophizing score for all participants. The Hospital Anxiety and Depression (HAD) questionnaire was used to quantify levels of anxiety and depression. The State-Trait Anxiety Inventory (STAI) was also used to determine the level of trait and state anxiety. Scores for early adverse life events was measured using Early Trauma Inventory (ETI). Somatization was determined using the Patient Health Questionnaire (PHQ). Menopause status in female subjects was assessed by a self-report question and missing values (30 female subjects) were imputed by considering subjects age ≤55 as premenopausal. **Table 3** provides additional demographic data about the subjects (i.e. ethnicity and education).

**Table 1:** Clinical variables for IBS patients.

	Type	Description	
		Female	Male
**Bowel Habit**	**Constipation**	26	3
	**Diarrhea**	19	5
	**Mixed**	20	4
	**Unspecified**	5	8
	**Total**	70	20
**Patient Variable**	**Two Sample T-Test**	**Mean(SD)**	**Mean(SD)**
**IBS Symptom Duration (year)**	p = 0.396	11.51(8.08)	11.12(7.42)
**Usual symptom severity (past week)**	p = 0.892	3.14(0.60)	3.17(0.62)
**Overall symptom severity (past week)**	p = 0.628	10.78(4.42)	11.40(4.58)
**Abdominal Pain (past week)**	p = 0.970	9.32(4.95)	9.375(5.19)

Groups: Healthy Control (HC), Irritable bowel syndrome (IBS).

**Table 2:** Clinical and Questionnaire Data.

		Male (N = 41)		Female (N = 225)	
Variable		Mean(SD)	p-value(df)	Mean(SD)	p-value(df)
**Age**	HC	34.04(11.23)	p = 0.350(39)	29.48(3.56)	p = 0.060(223)
	IBS	35.39(11.04)		31.61(9.01)	
**Early adverse life events** 1	HC	4.80(3.96)	p = 0.075(37)	3.51(3.56)	p = 0.041(208)
	IBS	6.98(5.24)		4.47(3.95)	
**Anxiety** 2	HC	3.19(2.94)	p = 0.007(36)	3.59(2.98)	p = 0.000(210)
	IBS	5.75(4.45)		5.36(3.25)	
**Depression** 3	HC	1.24(1.26)	p = 0.011(36)	1.40(2.04)	p = 0.000(210)
	IBS	3.00(3.52)		2.65(2.55)	
**Somatization** 4	HC	1.83(2.64)	p = 0.033(11)	2.21(1.82)	p = 0.000(143)
	IBS	5.43(3.55)		5.68(3.40)	
**Trait Anxiety** 5	HC	45.67(8.56)	p = 0.016(38)	43.94(8.93)	p = 0.000(221)
	IBS	53.05(12.32)		51.37(9.76)	
**Catastrophizing** 6	HC	0.19(0.39)	p = 0.000(36)	0.38(0.73)	p = 0.000(190)
	IBS	1.09(0.88)		1.21(1.08)	

Measures:

1 ETI global score;

2 HAD anxiety symptoms score;

3 HAD depression symptoms score;

4 PHQ score without IBS symptoms;

5 STAI raw score;

6 CSQ Catastrophizing score.

Groups: Healthy Controls (HC), Irritable bowel syndrome (IBS).

**Table 3:** Descriptive Demographic Data.

		Groups	Description		
			Female	Male	
**Ethnicity**					
	**White**	HC	67	9	
		IBS	44	15	
	**Asian**	HC	47	9	
		IBS	13	3	
	**Hawaiian**	HC	3	0	
		IBS	0	1	
	**Black**	HC	28	2	
		IBS	10	1	
	**American Indian**	HC	10	0	
		IBS	3	1	
	**TOTAL**	HC	155	20	
		IBS	70	21	
	**TOTAL**		**225**	**41**	
**Education**					
	**8^th^ Grade or Less**	HC	0	0	
		IBS	0	0	
	**Some High School**	HC	0	0	
		IBS	0	0	
	**High School** **Graduate**	HC	4	1	
		IBS	2	1	
	**Some College**	HC	56	7	
		IBS	18	7	
	**College Graduate**	HC	47	9	
		IBS	26	8	
	**Any Postgraduate** **Work**	HC	25	3	
		IBS	24	5	
	**Missing Data**		**23**	**0**	

Groups: Healthy Control (HC), Irritable bowel syndrome (IBS).

### Pipeline Workflows

Compete and automated Pipeline workflow protocols [27], [28] were used in this study to process this large scale of data in a timely fashion. Utilizing the LONI cranium grid server, which consists of 1,152 processors, the Pipeline workflow completed all the analysis in 6 days including the computationally intensive FreeSurfer surface modeling protocol [29], which normally takes 24 hours to complete one subject’s data. Workflows from the Pipeline library were configured for our data by specifying the data locations and all default parameters were used.

### MRI Acquisition

All brain images were collected at the UCLA Brain Mapping Center using one of the 4 structural acquisition sequences employed by the 14 study protocols. ANOVA indicated no differences in whole brain cortical thickness values were due to protocol. See **Table S1** for a more detailed description of the scan parameters.

### CT Analysis

All structural scans were first anonymized, converted from DICOM to analyzed format and then skull-stripped. Gray matter thickness was estimated by using a well validated method [29] implemented in FreeSurfer 4.0 [29] (available free to public, http://surfer.nmr.mgh.harvard.edu/fswiki and http://ucla.in/xSQPqT). The obtained CT maps were registered to ICBM brain surface and then vertex-wise correspondences were established between all cortical surface models using a Conformal Metric Optimization method [30]. An 8 mm heat kernel [31] was applied to smooth the realigned thickness maps to increase statistical power. Preprocessing and CT estimation were carried out using the LONI Pipeline [17] environment. A separate tissue-classification pipeline workflow (http://ucla.in/wxqlcJ) was employed to obtain total gray matter volume (TGMV), which was used as a covariate in the post hoc mapping and quantitative analysis.

### Whole Brain Analysis

SurfStat (http://www.math.mcgill.ca/keith/surfstat/) [32] was used to complete the whole brain CT analysis. A general linear model (GLM) was applied to identify any vertex-level CT changes associated with age and TGMV. Correction for multiple comparisons was accomplished using random field theory-based cluster analysis method [33]. A threshold false discovery rate (FDR) [34], [35] of 0.05 was used throughout the paper unless otherwise stated. For each subject’s cortical model, there were a total of 50,002 vertices (homologous across subjects) within each brain hemisphere.

### Region of Interest Analysis

A region-of-interest (ROI) approach was employed to detect bilaterally any subregional thickness change in the insula (INS) regions and the anterior cingulate cortices (ACC). These regions were further divided into sub-regions to spatially localize any effects. In each hemisphere, the insular sub-regions include anterior INS (aINS), mid INS (midINS) and posterior INS (pINS). ACC sub-regions include subgenual ACC (sgACC), pregenual ACC (pgACC), anterior mid cingulate (aMCC) and posterior mid cingulate (pMCC). These sub-regions were manually delineated on the 3D ICBM brain atlas by two well-trained technicians with good command of neuroanatomical knowledge. The 3D ROI masks were mapped back onto the ICBM surface space based on their Euclidean coordinates. Within each ROI and hemisphere, analysis of variance (ANOVA) was performed to test the significance of group differences while using age and TGMV as covariates. All reported p values were corrected for multiple comparisons using FDR. To quantify the structural differences, we calculated Cohen’s effect size d, reflecting structural differences in the scale of standard deviation units and values are interpreted as low (d = .20), moderate (d = .50), and high (d = .80).

### Correlation Analysis of Clinical Variables

To examine the possible links between brain structural change and the IBS phenotype, we assessed the association between all available clinical parameters and local cortical. The partial correlation coefficient was employed to test the significance of the association while accounting for the effect of age and TGMV. This analysis was completed separately for the males and females in the IBS cohort voxel-by-voxel within each hemisphere and ROI. All reported p values were corrected for multiple comparisons using FDR.

## Results

### Subject Characteristics

Demographic, clinical and behavioral variables of the sample are summarized in **Tables 1**, **2** and **3**. There were no significant age differences between HC and IBS subjects in male or female subgroups. The female sample was predominantly premenopausal = 147 (94.8%) of 155 HCs and 66 (94.3%) of 70 IBS. IBS patients as a group reported an average symptom duration of 11.5 years, an overall IBS symptom severity during the past week of 9.4 (on a scale from 0 to 20), and an abdominal pain rating of 8.4 (on a scale from 0 to 20). No sex related differences in these parameters were observed. In both subgroups, patients reported higher scores for symptoms of anxiety and depression (within normal range), trait anxiety (within normal range), somatization and catastrophizing. Female patients reported higher scores on early adverse life events.

### Whole Brain CT GLM Analysis

#### Main effect of group and sex

In the combined male and female sample, no significant main effect for group was observed between IBS and HC groups after FDR corrections. Significant main effect for sex after FDR was observed between female and male subjects in bilateral superior frontal gyrus (BA 8), left middle frontal-orbital gyrus, right superior temporal gyrus (BA 41 and 42), and bilateral superior frontal cortex (BA 8), with women showing greater CT in these regions.

#### Interaction of group and sex

**Figure 1:**
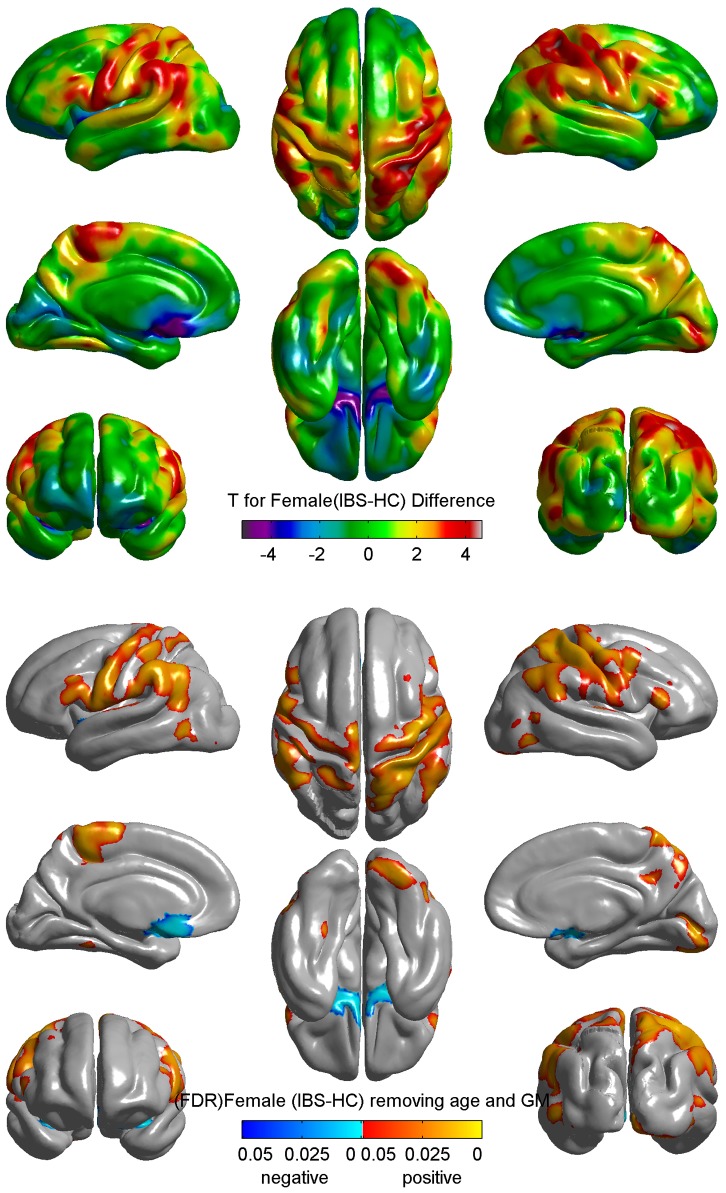
Differences in Cortical Thickness between Female IBS and Female HC. Upper: difference between female IBS and female controls from whole brain grey matter thickness analysis projected on average surfaces. T maps (uncorrected) were color coded: orange/red = thickening; blue/purple = thinning. Green = no change. Lower: FDR corrected p map from whole brain grey matter thickness analysis. Orange/red = female>male; blue/cyan = female<male. Cortical GM thickness was reduced in bilateral subgenual ACC and increased in bilateral motor and somatosensory cortex after controlling age and total grey matter volume. Groups: Healthy Control (HC); Irritable bowel syndrome (IBS).

The result for GLM analysis for the group difference in female subjects is shown in **Figure 1**. Compared to female HCs, female patients showed significant greater cortical thickness (FDR-corrected p<0.05) in pre and post central gyrus (Brodmann areas 1, 2, 3 and 4) and significantly smaller CT in bilateral subgenual ACC (BA 25) (**Figure 1**). These differences were not seen in male subjects.

### Regional CT Changes in INS and ACC Subregions using ROI Analysis

**Figure 2:**
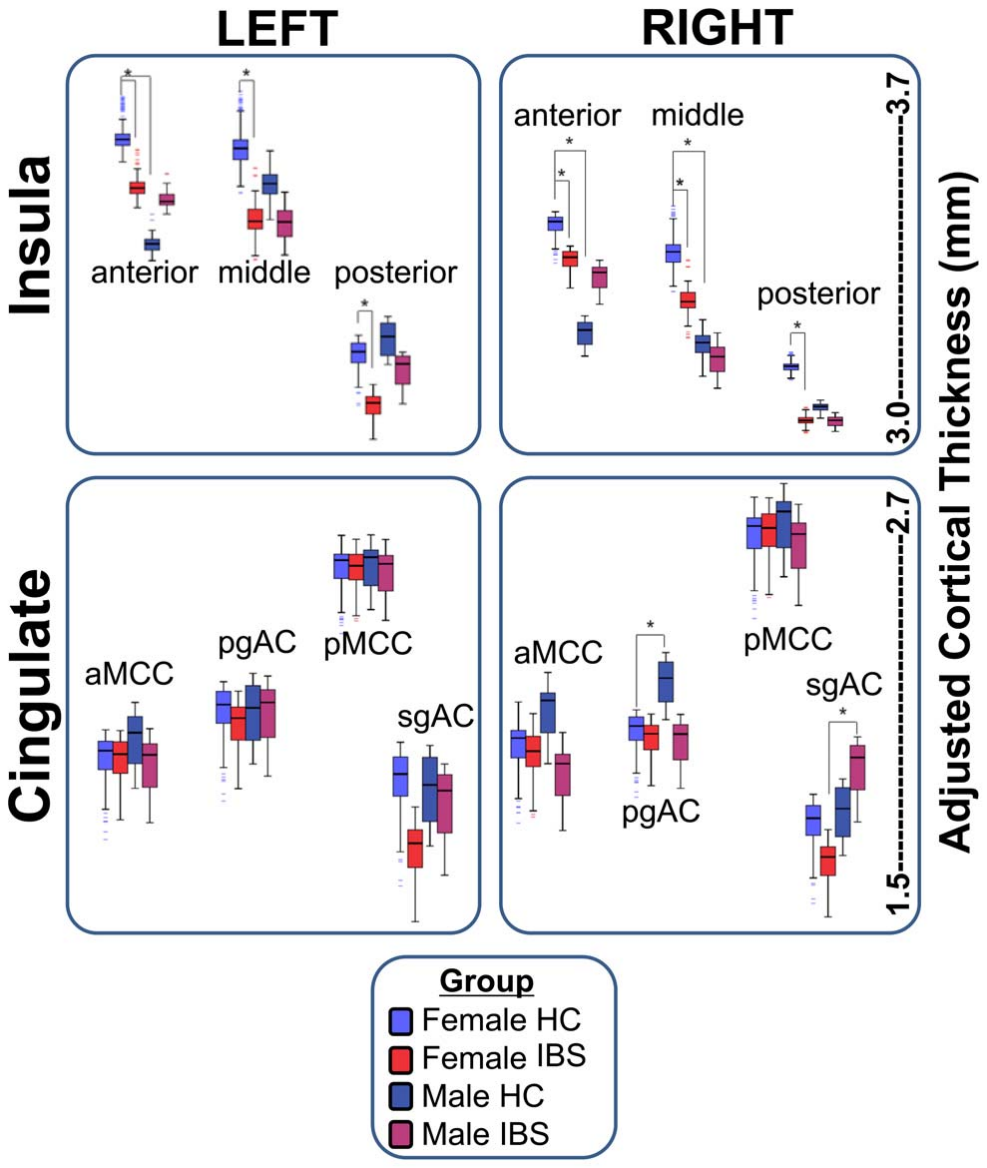
Cortical thickness sub-group Comparison. Cortical thinning in left aINS, left pINS, left pINS, left sgACC, right midINS, right pINS were only observed in female subjects. We also found that significant difference of cortical thickness in left aINS, rightaINS, right midINS and right pgACCin Female and Male HC. Another interesting finding is cortical thickness in right pgACC is significantly higher in male than female IBS patients. Groups: Healthy Control (HC); Irritable bowel syndrome (IBS).

Four contrasts were used to examine group and sex interactions, and all comparisons were corrected using FDR (0.05). Regional means and standard deviations as well as Cohen’s effect size differences are presented in **Table 4** and **Figure 2**.

**Table 4:** Regional means, standard deviations and Cohen’s effect size differences between groups.

Region	Hemisphere	Male HC	Female HC	Male IBS	Female IBS	Female IBS vs. Male IBS	Female HC vs. Male HC	Female IBS vs. Female HC	Male IBS vs. Male HC
		N = 21	N = 155	N = 20	N = 70				
aINS	Left	M = 3.296 (.387)	M = 3.574 (.352)	M = 3.406 (.373)	M = 3.441 (.286)	es = .10	es = .80	es = .40	es = −.30
	Right	M = 3.153 (.334)	M = 3.436 (.314)	M = 3.289 (.336)	M = 3.339 (.309)	es = .20	es = .90	es = .30	es = −.40
mINS	Left	M = 3.460 (.347)	M = 3.577 (.427)	M = 3.376 (.403)	M = 3.390 (.323)	es = .10	es = .30	es = .50	es = .20
	Right	M = 3.165 (.412)	M = 3.411 (.361)	M = 3.152 (.341)	M = 3.277 (.373)	es = .40	es = .70	es = .40	es = .04
pINS	Left	M = 3.072 (.257)	M = 3.044 (.319)	M = 3.001 (.278)	M = 2.912 (.268)	es = −.30	es = −.10	es = .40	es = .30
	Right	M = 2.989 (.283)	M = 3.099 (.325)	M = 2.956 (.331)	M = 2.963 (.319)	es = .02	es = .40	es = .40	es = .10
sgACC	Left	M = 1.764 (.381)	M = 1.787 (.380)	M = 1.713 (.347)	M = 1.624 (.344)	es = −.30	es = .10	es = .40	es = .10
	Right	M = 1.745 (.378)	M = 1.697 (.332)	M = 1.787 (.255)	M = 1.611 (.397)	es = −.50	es = −.10	es = .20	es = −.10
pgACC	Left	M = 1.934 (.245)	M = 1.939 (.307)	M = 1.917 (.253)	M = 1.897 (.224)	es = −.10	es = .02	es = .10	es = .07
	Right	M = 2.007 (.224)	M = 1.892 (.291)	M = 1.866 (.209)	M = 1.867 (.299)	es = .01	es = −.40	es = .10	es = .70
aMCC	Left	M = 1.830 (.268)	M = 1.786 (.359)	M = 1.762 (.408)	M = 1.781 (.404)	es = .10	es = −.10	es = .01	es = .20
	Right	M = 1.914 (.235)	M = 1.828 (.313)	M = 1.751 (.263)	M = 1.809 (.277)	es = .20	es = −.30	es = .10	es = .70
pMCC	Left	M = 2.289 (.162)	M = 2.288(.206)	M = 2.266 (.186)	M = 2.288 (.249)	es = .10	es = −.01	es = .00	es = .10
	Right	M = 2.404 (.163)	M = 2.378 (.196)	M = 2.342 (.172)	M = 2.384 (.168)	es = .30	es = −.10	es = −.03	es = .40
Postcentral gyrus(BA 1, 2, 3)	Left	M = 2.123 (.122)	2.011 (.179)	2.120 (.150)	2.122 (.150)	es = .01	es = −.70	es = −.70	es = .01
	Right	2.111 (.132)	1.969 (.203)	2.103 (.162)	2.097 (.195)	es = −.03	es = −.70	es = −.60	es = .10
Precentral gyrus (BA 4)	Left	2.368 (.238)	2.333 (.226)	2.462 (.183)	2.445 (.199)	es = −.10	es = −.20	es = −.50	es = −.50
	Right	2.325 (.234)	2.273 (.223)	2.421 (.181)	2.388 (.219)	es = −.20	es = −.20	es = −.50	es = −.50
Superior frontal gyrus(BA 8)	Left	M = 2.705 (.180)	M = 2.801 (.205)	M = 2.709 (.129)	M = 2.807 (.182)	es = .60	es = .50	es = −.03	es = −.02
	Right	M = 2.785 (.166)	M = 2.879 (.196)	M = 2.801 (.166)	M = 2.870 (.182)	es = .40	es = .50	es = .05	es = −.10
Middle frontal orbitalgyrus	Left	M = 2.495 (.189)	M = 2.557 (.194)	M = 2.474 (.169)	M = 2.558 (.165)	es = .50	es = .30	es = −.01	es = .10
	Right	M = 2.469 (.168)	M = 2.545 (.182)	M = 2.481 (.154)	M = 2.558 (.154)	es = .10	es = .40	es = −.10	es = −.10
Superior temporal gyrus(BA 41, 42)	Left	M = 2.661 (.212)	M = 2.603 (.336)	M = 2.806 (.205)	M = 2.699 (.262)	es = −.40	es = −.20	es = −.10	es = −.70
	Right	M = 2.625 (.190)	M = 2.523 (.326)	M = 2.665 (.226)	M = 2.591 (.258)	es = −.30	es = −.30	es = −.20	es = −.20

Groups: Healthy Control (HC), Irritable bowel syndrome (IBS).

Regions: aINS: anterior insula; mINS: middle insula, pINS: posterior insula; sgACC: subgenual anterior cingulate cortex; pgACC: pregenual cingulate cortex; aMCC: anterior mid cingulate cortex; pMCC: posterior mid cingulate cortex.

M: mean, es: effect size.

#### Sex related group differences between IBS and HCs

In female subjects, significantly smaller CT was observed for IBS compared to HCs in bilateral anterior insula (aINS; Cohen’s d = .40, .30), middle insula (midINS; d = .50, .40), posterior Insula (pINS, d’s = .40) and left sgACC (d = .40). In order to determine if bowel habits are related to the observed CT findings, we carried out a post-hoc comparison of differences in CT between female patients with constipation compared to those with diarrhea. Significant CT difference were only observed in the right pINS (p = 0.030, d = .70). In male subjects, trends indicated increased CT in the precentral gyrus (d = .50) and decreased CT in the anterior cingulate cortex, with effect sizes as big as d = .80 for right aMCC and R pgACC), but none of these changes reached statistical significance.

#### Sex related differences within group

In IBS subjects, significantly greater CT was observed in right sgACC in males compared to females. In HCs, significantly greater CT was observed in right pgACC in males compared to females. In addition, significantly smaller CT was observed in bilateral aINS in males compared to females.

### Correlation of CT Changes with Behavioral and Clinical Variables

#### Whole brain correlation analysis

**Figure 3:**
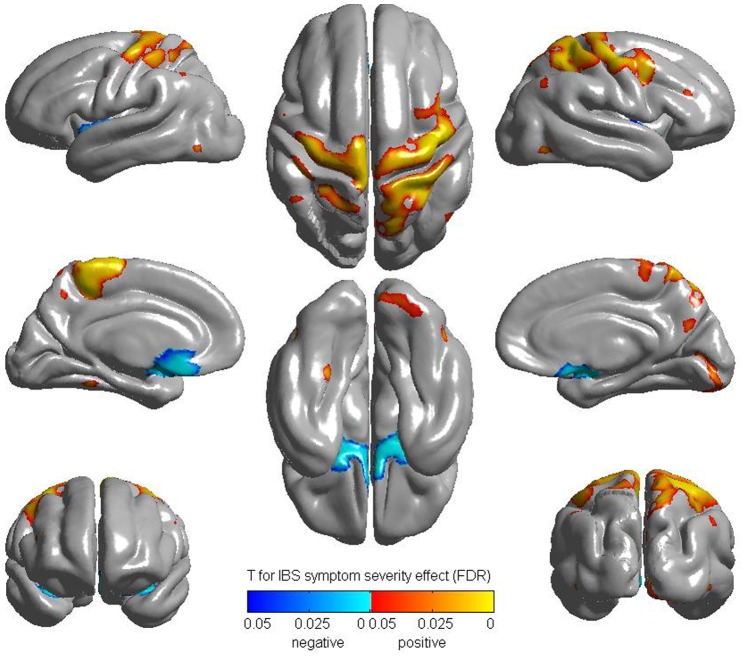
Correlation with BSQ Severity (FDR corrected). BSQ: Bowel symptom questionnaire.

As shown in **Figure 3**, the whole brain correlation analysis with BSQ_severity showed a positive correlation in the bilateral pre and post central gyrus, and a negative correlation in the bilateral sgACC in female IBS. When correlated to overall IBS symptom severity and abdominal pain ratings, a similar positive correlation was observed with bilateral precentral gyrus (abdomen region) and negative correlation with bilateral sgACC in female IBS. No significant correlations were observed between other variables including symptom duration and CT in female IBS patients. No statistically significant correlations were found in male IBS patients.

#### ROI correlation analysis

Partial correlation analysis of CT and various measures of symptom severity, duration and anxiety symptoms were performed on 6 separate ROIs and the resulting probability values were FDR corrected.

In female IBS, significant moderate negative CT correlations in the insula subregions were observed. Longer duration was found to be significantly correlated with reduced CT in right midINS (r = −0.293; p = 0.015). Higher anxiety symptom scores were significantly associated with reduced CT in right aINS (r = −0.273, p = 0.024) and right midINS (r = −0.248, p = 0.041). No significant correlations with IBS symptom severity were detected.

In male IBS, more clinical variables showed statistically significantly correlations with regional CT findings. More severe IBS symptom showed significant correlations with reduced CT in several INS subregions (left midINS r = −0.673; p = 0.002; right aINS, r = −0.597; p = 0.009; right midINS, r = −0.478; p = 0.045; and right pINS, r = −0.521; p = 0.027). Elevated trait anxiety (Tanxiety) was found to be significantly correlated with reduced CT in sgACC regions (r = −0.481; p = 0.044), sgACC (r = −0.525; p = 0.025), pgACC (r = −0.476; p = 0.046), and with increased CT in the right pINS (r = 0.469; p = 0.050). Higher scores for history of early adverse life events (ETI_Global) correlated significantly with increased thickness in left midINS (r = 0.556; p = 0.020).

## Discussion

Neuroplastic brain changes have been reported in many chronic pain syndromes, including IBS [3], [5], and often comorbid persistent pain conditions such as chronic pelvic pain [36], fibromyalgia [37], temporomandibular disorder [38], and vulvodynia [6]. While the majority of these studies reported decreases in regional CT and GM density, some also showed evidence for regional increases in CT involving S1, S2, and prefrontal regions [2], [39]. However, the great majority of studies were performed in small heterogeneous samples, and little attention has been given to possible influence of sex on the observed changes. Based on a review of the literature and limited examples of reversal of the structural brain changes with reversal of the chronic pain conditions, it has been suggested that some of the observed brain changes may be a consequence rather than the cause of the chronic pain conditions [4].

The current study reports on CT differences between a large, well phenotyped group of male and predominantly premenopausal female IBS and HC subjects, with a particular emphasis on sex differences. The main findings of the study are: 1) Within female subjects, IBS showed decreased CT in sgACC, and bilateral INS subregions, and increased CT in somatosensory and primary motor cortex when compared to HCs. 2) In contrast, no significant disease related differences in CT were observed within the male subjects, or between the combined male and female groups. However, in the HC group, male compared to female subjects showed smaller CT in the aINS, and greater CT in the pgACC. 3) Significant correlations between several measures of symptom severity and anxiety symptoms in the IBS group were observed, while correlations with symptom duration were weak or absent. To our knowledge, this is the first report on sex dependent, and symptom correlated increases and decreases in CT within somatosensory and viscerosensory brain regions obtained in a large sample of well phenotyped patients suffering from persistent abdominal pain.

### Novelty of Combining and Analyzing Data from Individual Studies

We demonstrated widespread regional increases and decreases in CT in IBS patients from 14 individual studies, and some of the changes were consistent with those reported in previous studies. Meta-analytic efforts usually offer limited interpretation due to small statistical power, small reliability of findings, and non-representativeness of samples, but aggregating data across multiple studies through mega-analyses has accelerated progress in mapping brain structure and function by improving these limitations [40]. Although between-study factors may be a source of variability, results that generalize over multiple studies are considered more reliable and less likely to be related to the characteristics of a single study’s design.

### Neuroplastic Brain Changes in Female IBS Patients

The current study is consistent with earlier reports in smaller samples of patients with various chronic pain conditions, showing GM reductions in ACC and INS subregions [41], [42]. Two previous studies have reported GM changes in premenopausal female IBS patients (of similar age as those in the current study), using voxel based morphometry and CT analysis. In a small sample of patients (n = 16), Blankstein et al. [5] reported cortical and subcortical changes in IBS, including thinning in midcingulate cortex compared to HCs. However, this change was not associated with clinical or behavioral parameters. In a larger study (n = 55), Seminowicz et al. [3] reported widespread GM changes in cortical and subcortical brain regions (including prefrontal and posterior parietal cortex) which were only seen in the voxel-based morphometry, but not in the cortical thickness analysis [3]. In this study, trends for increased GM were found in viscera and somatosensory regions (pINS, S2), while no GM reductions in ACC or INS were observed. The reasons for the differences between the two studies and the current results remains unclear, but are likely to include sample size, patient characteristics (including possible differences in medications), use of different statistical algorithms and templates, increased power in the larger sample to detect moderate effect size differences, rigorous error control applied in the present study, and decreases in variability in the sampling mean estimates afforded by increased sample size.

The current study demonstrates significantly greater CT in female patients in S1, a brain region involved in sensory discriminative processing of both noxious and innocuous stimuli [43], [44], [45]. Based on both symptom reports [46], and results from quantitative pain testing, female IBS patients show evidence for widespread somatic hyperalgesia, including heat pain stimuli [47], [48], an observation consistent with the observed changes in somoatosensory cortex. In addition, female IBS patients are more likely to report comorbidity with other pain syndromes (such as IC/PBS, FM and chronic fatigue syndrome) [49], [50], and report more extraintestinal symptoms [51].

Currently available data do not permit to address the causality of these findings. On the one hand, GM increases in S1 and ACC in response to repetitive noxious stimulation have recently been reported in HCs [52], suggesting that differences in S1 and ACC can be a consequence of repeated noxious insults, possibly reflecting the observed habituation of subjects to the repeated stimulus. On the other hand, a positive correlation between CT in S1 and pain and temperature sensitivity has been reported in HCs without any pain history or stimulus exposure, including a negative correlation of temperature sensitivity with CT in the anterior midcingulate cortex. [53] These findings strongly suggest that such regional GM variations are not necessarily dependent on the presence of a chronic pain conditions or ongoing nociceptive input, but could also be influenced by genetic or epigenetic factors [54], [55], and in the case of IBS may be a preexisting abnormality. The fact that the majority of CT differences including the somato and viscerosensory regions (with the exception of CT in the midINS) were not significantly correlated with symptom duration supports such a hypothesis. Future studies in pediatric populations, longitudinal treatment studies, and identification of possible correlations with genetic factors are needed to further test this hypothesis. On the other hand significant correlations of the observed CT changes were observed with several measures of symptom severity and a measure of anxiety. For example, reported symptom severity was positively correlated with the CT increases in bilateral pre and post central gyrus, and negatively with the reductions in sgACC. In contrast, the observed CT reductions in the right aINS were strongly positively correlated with reported anxiety symptoms severity. The aINS represents an interoceptive association cortex with inputs from prefrontal cortex and the amygdala, and activity in this region has been associated positively to anxiety sensitivity [25].

The mechanisms underlying the observed increases and decreases in CT remain to be determined, even though various molecular mechanisms have been proposed [56], [57]. It is likely that the regional increases and decreases in GM are driven by different mechanisms, which may include neuronal or glial cell loss, and changes in the density of dendritic spines [58]. The latter mechanism has been demonstrated to underlie stress induced reductions in hippocampal volume in animal models where it is modulated by female sex hormones and is reversible under certain conditions [59].

### Sex related Differences in Neuroplastic Changes

Sex related differences in CT were observed both in the HCs and in the IBS group. Amongst the HCs, males compared to females showed greater CT in the pregenual cingulate cortex, and smaller CT in bilateral aINS. These findings are similar to some previously published studies of sex effects on neuroplasticity [60], [61].

Surprisingly, in contrast to female IBS patients, male patients did not show statistically significant CT differences when compared to their healthy counterparts and amongst IBS subjects, males showed greater CT in sgACC, compared to females. One possible explanation for the lack of significant differences is the smaller sample size, which is also suggested by the observed non-significant trends in the male IBS patients. However, as the majority of previously published studies included only females or mixed samples, these findings for the first time suggest that neuroplastic changes associated with persistent abdominal pain may differ between male and female patients. Using diffusion tensor imaging (DTI), sex related differences in the white matter integrity (as measured by lower fractional anisotropy) has recently been described in a large sample of IBS patients [62]. In this study, female patients showed lower fractional anisotropy within the globus pallidum, regions of the thalamus, and primary sensory and motor regions compared to male IBS subjects. When viewed together with the findings of the current study in pre and postcentral gyrus, they support the concept that there is a fundamental reorganization of pathways in the brain that are involved in processing and integration of sensory information, and that these changes are predominantly seen in female patients. Previous studies have shown that female IBS patients report more symptoms suggestive of sensory amplification [51].

The absence of significant IBS related differences when the combined male and female samples were analyzed together, is presumably related to the fact that only female patients showed significant disease related differences.

### Limitations

There are several limitations of this study. Pooling of data from multiple studies may have contributed some heterogeneity to the sample. The lack of significant CT changes observed in male patients may in part be related to the difficulty in recruiting subjects and the smaller sample size (n = 41) compared to female patients (n = 225). For example, a sample size of 52 males (26 IBS) would be needed to detect a significant effect size difference of d = .70 as was observed in the pgACC with 80% power based on an independent sample t-test. Similarly, 51 males per group would be needed to detect effect size differences as big as d = .50 which was the observed effect size increase in the supplementary motor cortex in IBS males compared to HCs. Some of the observed differences may be due to the intake of centrally acting medications in the IBS group, in particular tricyclic antidepressants. Patients on a stable dose (3 months) of such medications were allowed in the study, but incomplete records of such intake prevented us from assessing possible medication effects on our findings. Lastly, we were unable to assess possible effects of female sex hormones on brain responses in our sample of predominantly premenopausal females, scanned during the follicular phase of the menstrual cycle.

### Summary and Conclusions

To our knowledge, this is the first study reporting sex related regional differences in cortical thickness in a large sample of IBS patients. While reductions in cortical thickness were observed in affect related regions, increased cortical thickness was seen in sensory and motor regions, and these disease related differences were only seen in female patients. When viewed together with sex related differences in prevalence [63], [64], presence of somatic and visceral comorbidities [15], functional [65], [66] and white matter abnormalities [36], [67], [68], these findings support the concept that different brain mechanisms underlie symptom generation in female and male patients. As previously suggested, symptoms of pain and discomfort in women may be related primarily to increased perception and facilitation of sensory signals, while in men, autonomic nervous system hyperresponsiveness may play a more important role.

### Note

As of September 2013, the Laboratory of Neuro Imaging (LONI) will be relocated to the University of Southern California (USC). Thus, some of the URL links, web-page references, and internet resources cited throughout this manuscript may be relocated to appropriate subdomains under http://www.loni.usc.edu. If you find broken links or defunct URLs please contact help@loni.usc.edu.

## Supporting Information

Figure S1 **Example Inspection and Validation of Regions.** Representative visual inspection and validation of labeling in sub-regions by a trained neuroanatomist. This figure shows rated insula surfaces for 3 subjects and the corresponding ICBM atlas surface. Insula sub-regions were color coded (Red = anterior insula,blue = mid insula;brown = posterior insula).
(TIFF)

Table S1 **Scan Parameters used in present aggregated data.** Summary of scan parameters from 4 different studies.
(DOC)
