# Supplementary figures and images for: Sex-Related Differences of Cortical Thickness in Patients with Chronic Abdominal Pain
Source: PLoS One. 2013 Sep 5;8(9):e73932. doi: 10.1371/journal.pone.0073932 (PMC3764047; doi:10.1371/journal.pone.0073932)

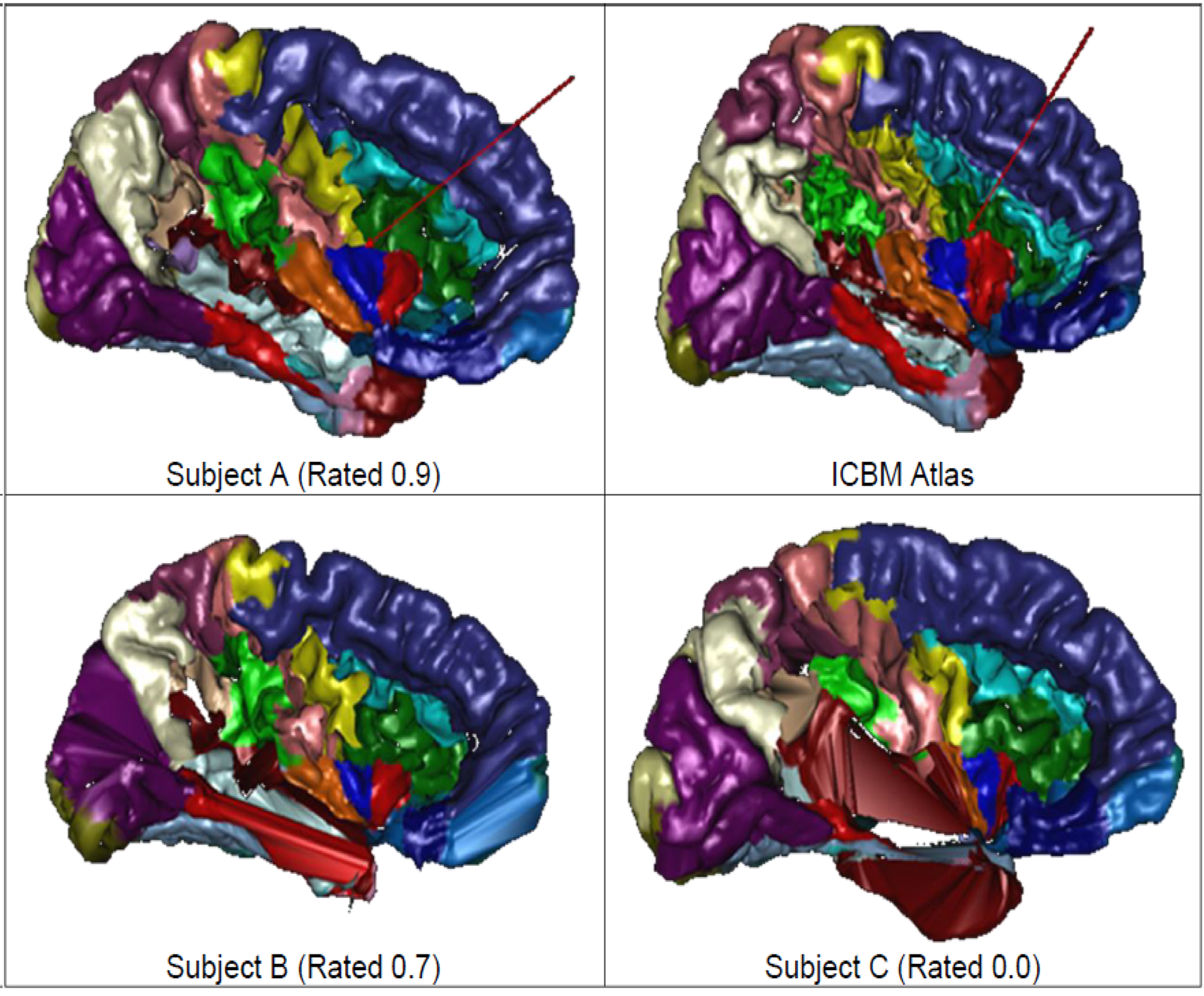

Supplement: Figure S1 — Example Inspection and Validation of Regions. Representative visual inspection and validation of labeling in sub-regions by a trained neuroanatomist. This figure shows rated insula surfaces for 3 subjects and the corresponding ICBM atlas surface. Insula sub-regions were color coded (Red = anterior insula,blue = mid insula;brown = posterior insula). (TIFF) [file pone.0073932.s001.tiff]
