# Supplementary material for: Sex-Related Differences of Cortical Thickness in Patients with Chronic Abdominal Pain
Source: PLoS One. 2013 Sep 5;8(9):e73932. doi: 10.1371/journal.pone.0073932 (PMC3764047; doi:10.1371/journal.pone.0073932)
Supplement: Table S1 — Scan Parameters used in present aggregated data. Summary of scan parameters from 4 different studies. (DOC) [file pone.0073932.s002.doc]

Table S1: Scan parameters used in present aggregated data

| SEQUENCE | TR(ms) | TE(ms) | Flip Angle(°) | Scanner | Strength(T) | Studies | Number of Scans | | | |
| --- | --- | --- | --- | --- | --- | --- | --- | --- | --- | --- |
|  |  |  |  |  |  |  | HC | | IBS | |
|  |  |  |  |  |  |  | M | F | M | F |
| 1 | 20 | 3.39 | 25 | SIMENS TRIO | 3.0 | 1,2,4 | 0 | 55 | 0 | 14 |
| 2 | 2300 | 2.85 | 9 | SIEMENS SONATA | 1.5 | 3,5,7,8 | 13 | 33 | 13 | 43 |
| 3 | 1900 | 4.38 | 15 | SIMENS SONATA | 1.5 | 6,11 | 0 | 41 | 0 | 5 |
| 4 | 2200 | 3.26 | 9 | SIMENS TRIO | 3.0 | 9,10,12,13 | 8 | 26 | 7 | 8 |
